# Natural Shape-Retaining Microcapsules With Shells Made of Chitosan-Coated Colloidal Lignin Particles

**DOI:** 10.3389/fchem.2019.00370

**Published:** 2019-05-22

**Authors:** Tao Zou, Mika H. Sipponen, Monika Österberg

**Affiliations:** Department of Bioproducts and Biosystems, School of Chemical Engineering, Aalto University, Espoo, Finland

**Keywords:** lignin nanoparticles, chitosan, thin film coating, Pickering emulsion, microcapsule, drug delivery

## Abstract

Thin film coating of charged nanoparticles with oppositely charged polymers is an efficient and straightforward way for surface modification, but synthetic polyelectrolytes should be replaced by abundant biopolymers. In this study a thin film of chitosan was adsorbed onto colloidal lignin particles (CLPs) that were then systematically studied for olive oil stabilization with an objective to develop shape-retaining microcapsules that comprised of only renewable biomaterials. Full surface coverage was achieved with merely 5 wt% of chitosan relative to the dry weight of CLPs, reversing their surface charge from negative to positive. Such modification rendered the chitosan-coated particles excellent stabilizers for forming Pickering emulsions with olive oil. The emulsion droplets could be further stabilized by sodium triphosphate that provided ionic intra- and inter-particle cross-linking of the chitosan corona on the CLPs. Following the optimum conditions, the non-cross-linked microcapsules exhibited a strong stability against coalescence and the electrostatically stabilized ones additionally retained their shape upon drying and rewetting. Non-cross-linked microcapsules were used to demonstrate encapsulation and rapid release of ciprofloxacin as a model lipophilic drug in aqueous media. Overall, the combination of antimicrobial chitosan and antioxidative lignin nanoparticles hold unprecedented opportunities as biocompatible and biodegradable materials for controlled drug delivery.

## Introduction

Lignin is the second most abundant natural biopolymer and the most abundant aromatic polymer in nature (Ragauskas et al., 2014). Annually over 70 million tons of industrial lignin is produced as a by-product in the pulp/paper industry. However, only 5% of industrial lignin is commercialized as value-added products, such as additives, dispersants, adhesives, or surfactants (Laurichesse and Avérous, 2014). The rest 95% is used as low value energy source or simply treated as waste. The low extent of commercialization of lignin is mainly due to its complex and inhomogeneous structure resulting from different sources and extraction processes (Li et al., 2015). However, recent studies have shown that fabricating spherical micro- or nanoparticles from lignin not only enables the large scale utilization of lignin (Ago et al., 2016; Leskinen et al., 2017a; Ashok et al., 2018; Lintinen et al., 2018), but also improves properties such as surface activity, antioxidative, UV shielding, and antimicrobial activity for potential high-value applications (Beisl et al., 2017).

To name a few examples, colloidal lignin particles (CLPs) have been utilized to entrap hydrophobic drugs for drug delivery purposes in biomedicine (Chen et al., 2016; Dai et al., 2017; Figueiredo et al., 2017; Sipponen et al., 2018b). By introducing CLPs in cellulose nanofilms, Farooq, and co-workers rendered the composite films waterproof, provided antioxidant activity and UV-shielding in addition to the improved mechanical properties (Farooq et al., 2019). Surface-modification of CLPs by adsorption has been demonstrated with synthetic poly(diallyldimethylammonium chloride) (Lievonen et al., 2016), proteins (Leskinen et al., 2017b), and cationic lignin (Sipponen et al., 2017). Such modifications alter the interfacial properties of the particles and broaden the window for possible high-value applications. For instance, cationic CLPs were demonstrated for binding negatively charged enzymes for enhanced catalytic activity in aqueous ester synthesis (Sipponen et al., 2018a).

In addition, micro- and nanoscaled lignin particles have been exploited as surface-active stabilizers for Pickering emulsions (Wei et al., 2012; Yang et al., 2013; Nypelö et al., 2015; Ago et al., 2016; Sipponen et al., 2017). Pickering emulsions are stabilized by solid particles at the oil/water interface (Pickering, 1907). Compared to conventional surfactant-stabilized emulsions, Pickering emulsions are more stable against coalescence, because high energy input is required to remove solid particles from the oil/water interface (Binks, 2002; Rayner et al., 2014; Berton-Carabin and Schroën, 2015). Additionally, some food-grade particles are less toxic than synthetic surfactants (Yang et al., 2017). Therefore, Pickering emulsions can be applied in a wide range of fields including biomedicine, cosmetics, and food (McClements, 2015; Tang et al., 2015; Yang et al., 2017).

To date, lignin-stabilized Pickering emulsions reported in scientific articles are mainly aliphatic or aromatic hydrocarbon oil-based, e.g., kerosene, hexadecane, styrene, and toluene (Wei et al., 2012; Yang et al., 2013; Nypelö et al., 2015; Ago et al., 2016; Sipponen et al., 2017). Such oils are often toxic, which therefore are not suitable for biomedicine, cosmetics, or food. Instead, triglyceride vegetable oils are a better choice, since they do not present any safety issues (Pouton and Porter, 2008). Furthermore, triglycerides are good solvents for many lipophilic drugs and have the ability to increase intestinal wall permeability, which makes them particularly useful for drug delivery (Kalepu et al., 2013). Nevertheless, unmodified CLPs exhibit limited capacity in stabilizing triglyceride-in-water Pickering emulsions, possibly due to insufficient hydrophilicity. Sipponen et al. modified CLPs by adsorption of cationic lignin to form more hydrophilic cationic CLPs (c-CLPs), which exhibited improved capacity for stabilizing olive oil compared to unmodified CLPs (Sipponen et al., 2017). Yet, the formed olive oil-in-water emulsions were not sufficiently stable. Additionally, alike other quaternary ammonium substances (Zhang C. et al., 2015), the cationic lignin grafted with quaternary ammonium groups may be toxic.

Chitosan, on the other hand, has shown good emulsification capacity in stabilizing triglyceride-in-water emulsions whether in the form of molecules (Schulz et al., 1998; Del Blanco et al., 1999; Rodriǵuez et al., 2002) or particles (Mwangi et al., 2016a; Shah et al., 2016; Asfour et al., 2017). Furthermore, chitosan possesses biologically relevant properties such as biocompatibility, biodegradability, bio-adhesion, and antimicrobial activity (Pavinatto et al., 2010; Croisier and Jérôme, 2013; Asfour et al., 2017). As a consequence, chitosan appears to be a plausible material for modifying CLPs to form triglyceride-based Pickering emulsions.

In this work, we systematically studied the modification of CLPs by thin film coating of chitosan and determined the emulsification capacities of the chitosan-coated CLPs (chi-CLPs) for olive oil. We used sodium triphosphate (STP) to ionically cross-link the chi-CLPs locating at the oil-water interface and compared the mechanical properties of the electrostatically stabilized chi-CLP microcapsules to those of non-cross-linked ones. Finally, we demonstrated the ability of the chi-CLP microcapsules in the encapsulation and release of ciprofloxacin at various pH values at 37°C. Overall, this work provides optimum conditions for the preparation of non-coalescent and shape-retaining microcapsules for future applications in controlled drug delivery in the field of biomedicine.

## Materials and Methods

### Materials

Kraft lignin (BioPiva 100) used in this work was isolated from softwood using the LignoBoost® technology at Domtar's Plymouth plant (NC, USA). The kraft lignin was well-characterized in the previous publication (Sipponen et al., 2018a). The number and weight average molecular weights (M_n_ and M_w_) of the kraft lignin are 1,193 and 5,250 g/mol, respectively. The carboxyl groups, aliphatic hydroxyl groups, and total phenolic hydroxyl groups of the kraft lignin are 0.57, 1.89, and 4.05 mmol/g, respectively. Tall oil fatty acid (TOFA) “For2” was a kind gift from Forchem Oyj (Finland). Chitosan (molecular weight 100 to 300 kDa, deacetylation degree ≥90%) and sodium triphosphate (STP) (≥98%) were purchased from Fisher Scientific (Acros organics). Olive oil (highly refined, low acidity), ciprofloxacin (≥98%), poly(styrene) (${\bar{M}}_{w}$: 35,000 g/mol) and acetic acid (glacial, ≥99.8%) were purchased from Sigma-Aldrich. The content of free fatty acid in olive oil was determined to be 1.9 ± 0.2 wt% (*n* = 7) by titration (Sipponen et al., 2017). All purchased chemicals and solvents were used without further purification.

### Preparation of Chitosan Solution

1 wt% chitosan solution was prepared by dissolving 1 g of chitosan in 99 g of 0.1 M acetic acid under stirring for 24 h. The dissolution of chitosan in 0.1 M acetic acid resulted in an increase of the pH value from 2.9 to 4.5, which indicated a partial protonation of chitosan (ca. 60 to 70% protonation of the primary amine groups, calculated according to the pKa 4.75 of acetic acid and the deacetylation degree of chitosan).

### Preparation of Aqueous Colloidal Lignin Particles

Preparation of 0.2 wt% colloidal lignin particle (CLP) dispersion followed the procedure described in the previous publication (Sipponen et al., 2017) with the modification that in this study 2 g kraft lignin (dry weight) was dissolved in 200 g of acetone-water mixture (mass ratio: 3:1), instead of using THF-water mixture as the solvent. The final aqueous CLP dispersion (0.2 wt%) was obtained with a lignin mass yield of 85%. The particle diameter and zeta potential of the CLPs at native pH 3.9 were determined to be 97 nm (PDI 0.18) and −27 mV, respectively. The preparation of 1 wt% CLP dispersion was similar to that of 0.2 wt% CLP dispersion except that in this case rotary evaporation (40°C under reduced pressure) was used to remove acetone instead of dialysis. 0.5 wt% CLP dispersion was prepared by diluting the 1 wt% CLP dispersion with deionized water. The particle diameter and zeta potential of the CLPs (0.5 and 1 wt%) were 113 nm (PDI 0.19) and −30 mV (at native pH 3.1), respectively.

### Preparation of Chitosan-Coated Colloidal Lignin Particles (chi-CLPs)

Chitosan-coated colloidal lignin particles (chi-CLPs) were prepared by adding CLP dispersion (0.2 or 0.5 or 1 wt%) slowly into 1 wt% chitosan solution under vigorous stirring for 30 min. The prepared chi-CLP dispersions were stored over night before use. For 0.2 wt% chi-CLP dispersions, the mass ratio of chitosan to CLP was varied from 0 to 200 mg/g. For 0.5 and 1 wt% chi-CLP dispersions, chi-CLPs were prepared at a fixed mass ratio of 50 mg/g.

### Preparation of chi-CLP Stabilized Oil-in-Water Pickering Emulsions

The Pickering emulsions were prepared by ultrasonication (Branson 450 Digital Sonifier with a 3 mm-diameter microtip) under ice bath condition at a fixed volume ratio of 1:1 olive oil to chi-CLP (or CLP) dispersion. More specifically, 60 s with the cycles 10 s on and 5 s off were applied for emulsion formation, the amplitude was set at 10% for a total volume of 2 ml and 40% for 10 ml. These procedures resulted in similar size distributions of oil droplets when the concentration of the chi-CLP (50 mg/g) dispersion was 0.5 or 1 wt%.

### Preparation of Ionically Cross-Linked Pickering Emulsions

Pickering emulsion formed with 1 wt% chi-CLP (50 mg/g) dispersion was used for the cross-linking study. The preparation was achieved by adding the emulsion slowly into 6 wt% sodium triphosphate (STP) aqueous solution at the volume ratio of 1:9 (emulsion: STP solution) under vigorous stirring for 30 min.

### Preparation of Ciprofloxacin-Loaded Pickering Emulsions

1 wt% chi-CLP (50 mg/g) dispersion was used for forming the emulsion with ciprofloxacin-loaded oil for release study. Ciprofloxacin was firstly dissolved (20 mg/mL) in TOFA followed by dilution with olive oil to 2 mg/mL. The emulsion formation followed the aforementioned procedure.

### Ciprofloxacin Release Study

The release study was performed in three different pH buffers, pH 2 (the buffer was prepared by mixing 0.1 M KCl and 0.02 M HCl to obtain a solution at pH 2), pH 5.5 (0.05 M PBS) and pH 7.4 (0.05 M PBS) at 37°C. For each sample, 1.2 mL ciprofloxacin-loaded emulsion (50% oil phase) was injected into 60 ml of buffer solution. The aliquots were taken at various time intervals and filtered through a 0.2 μm syringe filter to separate the oil droplets and/or lignin particles. The concentration of ciprofloxacin in the buffer was calculated from the absorbance values at 277 nm, after correcting with the absorbance resulting from minor dissolution of lignin in the absence of ciprofloxacin, according to the calibration curve shown in Figure S1. The average of four ciprofloxacin-loaded samples and two reference samples were used in the analysis and reporting of data.

### Particle Diameter and Zeta Potential Analysis

Particle diameters and zeta potentials of CLPs and chi-CLPs were analyzed using a Zetasizer Nano ZS90 instrument (Malvern Instruments Ltd., U.K.). A dip cell probe was utilized for the determination of the zeta potential. CLP, chi-CLP dispersions and chitosan solution were diluted accordingly with deionized water or pH 4.5 acetic acid prior to measurement. Mean values of three replicates of the particle diameter (Z-average, intensity mean) and zeta potential were used in the analysis and reporting of data.

### Droplet Diameter and Uniformity of Emulsion Analysis

The droplet diameter of the emulsion was determined by static light scattering (Mastersizer 2000, Malvern, UK). The emulsions were diluted with deionized water to reach the laser obscuration of 6 to 12% prior to starting the measurement. The refractive index (RI) of olive oil and water used in the calculations were 1.47 and 1.33, respectively. Mean droplet diameter was calculated over volume data (d_43_, De Brouckere Mean Diameter). Uniformity of the droplets was calculated according to Equation (1)

(1) $$\begin{array}{r} Uniformity=\;\frac{\sum X_{i}|d\left(v,\;0.5\right)-d_{i}|}{d{\left(v,\;0.5\right)}_{i}} \end{array}$$

where *d*(*v*, 0.5) is the median diameter in the volume-based distribution, *d*_*i*_ is the diameter in class *i* and *X*_*i*_ is the corresponding volume fraction in %. Mean values of six replicates of mean droplet diameter (d_43_) and uniformity were used in the analysis and reporting of data.

### Microscopy Observation

#### Confocal Microscopy

The emulsions stabilized by 1 wt% chi-CLP (50 mg/g) were imaged with the confocal laser scanning microscope (Leica DMRXE, Germany). Emulsions were diluted 50 times with deionized water or with 6 wt% STP aqueous solution followed by staining the oil with Nile red (1 mg/mL in ethanol) (ca. 50 μl of Nile red in 1 ml emulsion) prior to measurement. For each sample, a drop (5 μl) of the Nile red-stained emulsion was placed on the glass slide for imaging at the wavelength of 488 nm, using a 10 × air objective and a 63 × oil immersion objective.

#### Optical Microscopy

A Leica Zeiss (DM750) optical microscope was used for observing the emulsions without staining.

#### Environmental-SEM

A Zeiss Environmental Scanning Electron Microscope (EVO HD15) was used for observing the surface morphology of the oil droplets. The emulsion capsule that was stabilized with 1 wt% chi-CLP (50 mg/g) and cross-linked with STP was selected for the observation. A drop (5 μl) of the diluted emulsion (500 times with 6 wt% STP aqueous solution) was cooled at −20°C in a Peltier cooling element in order to freeze the oil and sublimate the water. During the observation, the temperature was kept at −20°C and the pressure of the SEM chamber was set at 50 Pa, images were captured with an EPSE detector.

#### AFM

The AFM analysis was carried out with a MultiMode eight atomic force microscope equipped with a NanoScope V controller (Bruker Corporation, U.S.A.). The images for CLPs were obtained in tapping mode under ambient air condition with NCHV-A tapping mode probes (Bruker). The images for oil droplets (stabilized by chi-CLP and cross-linked with STP) were obtained in ScanAsyst mode under ambient air condition with SCANASYST-AIR probes (Bruker). Samples were prepared by dropping 5 μl of the diluted dispersion or emulsion on the mica surfaces and drying under ambient conditions. Nanoscope analysis 1.5 software was used for image analysis.

### QCM-D

The lignin substrates for QCM-D studies were prepared as described by Salas et al. (2013) except that the lignin used in this study was kraft lignin. In brief, lignin was spin-coated onto gold-coated QCM crystals (Q-Sense, Sweden) that had been pre-coated with poly(styrene). Adsorption of chitosan on the thin films were carried out using QCM-D E4 (Q-Sense, Sweden) in continuous flow mode. Chitosan was firstly dissolved (1 wt%) in 0.1 M acetic acid and then diluted with pH 4.5 acetic acid to 100 μg/mL. During the measurements, the lignin films were firstly rinsed with pH 4.5 acetic acid buffer at the flow rate of 0.1 mL/min until a plateau baseline (Δ*f*_5_) was reached, followed by replacing the acetic acid buffer with the diluted chitosan solution. The temperature was controlled at 25°C throughout the experiments. The mass of chitosan adsorbed on the lignin surface was related to the change of resonance frequency Δ*f* according to the Sauerbrey equation (Sauerbrey, 1959):

(2) $$\begin{array}{r} \text{Δ}\text{m}=\;-\text{CΔf}/\text{n} \end{array}$$

where Δm is the change of mass, Δf is the change of resonance frequency determined by the device, C is a constant (0.177 mg m^−2^ Hz^−1^) that describes the sensitivity of the device to changes in mass, n is the overtone number (*n* = 1 represents the fundamental frequency at 4.95 MHz). The energy dissipation due to adsorption is indicated by the dissipation factor D (Rodahl and Kasemo, 1996), which is defined as:

(3) $$\begin{array}{r} D=\frac{E_{dis}}{{2\pi E}_{st}} \end{array}$$

where E_dis_ is the dissipated energy and E_st_ is the stored energy during one oscillation cycle. The dissipation change is defined as, Δ*D* = *D*–*D*_0_, where *D*_0_ denotes the initial dissipation prior to adsorption. In this work, the average values (Δ *f*_5_ and Δ*D*_5_) of two replicate samples were used in the analysis and reporting of data.

## Results and Discussion

**Figure 1:**
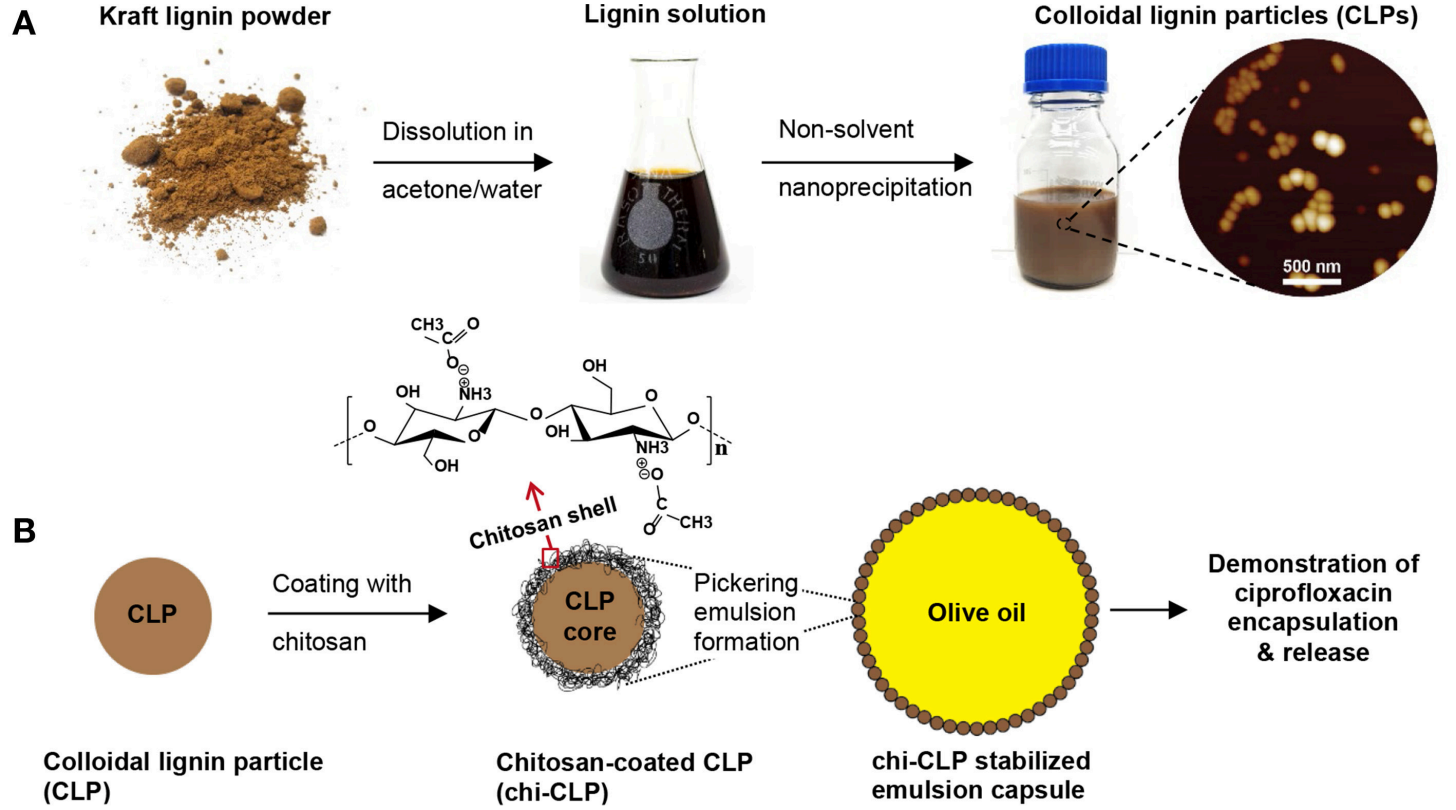
General scheme of this work. **(A)** Preparation of colloidal lignin particles (CLPs). **(B)** Coating of CLPs with chitosan for forming Pickering emulsion with olive oil, and demonstration of the emulsion capsules for drug delivery. Note: not drawn to scale.

The overarching objective of this work was to establish a reliable approach for chitosan-coated CLPs, and application of the modified particles in stabilization of olive oil-in-water Pickering emulsion capsules. A general scheme of this work is shown in Figure 1. The CLPs were prepared by rapid anti-solvent nanoprecipitation method, followed by either dialysis or evaporation to remove the acetone that was used as solvent. In contrast to the more commonly used THF (Lievonen et al., 2016), acetone can be easily removed by evaporation, as acetone has no azeotrope with water (Smallwood, 2012). The obtained CLPs were modified with thin film coating of chitosan by physical adsorption and systematically studied for the stabilization of Pickering emulsions. Selected emulsion capsules were further stabilized by non-covalent cross-linking. Encapsulation of a model drug into the oil phase and its release from the non-cross-linked capsules was finally demonstrated in aqueous media. The first important step was to optimize the chitosan coating and emulsion formation processes, which are discussed more in detail below.

### Determining the Minimum Needed Chitosan Coating on CLPs

**Figure 2:**
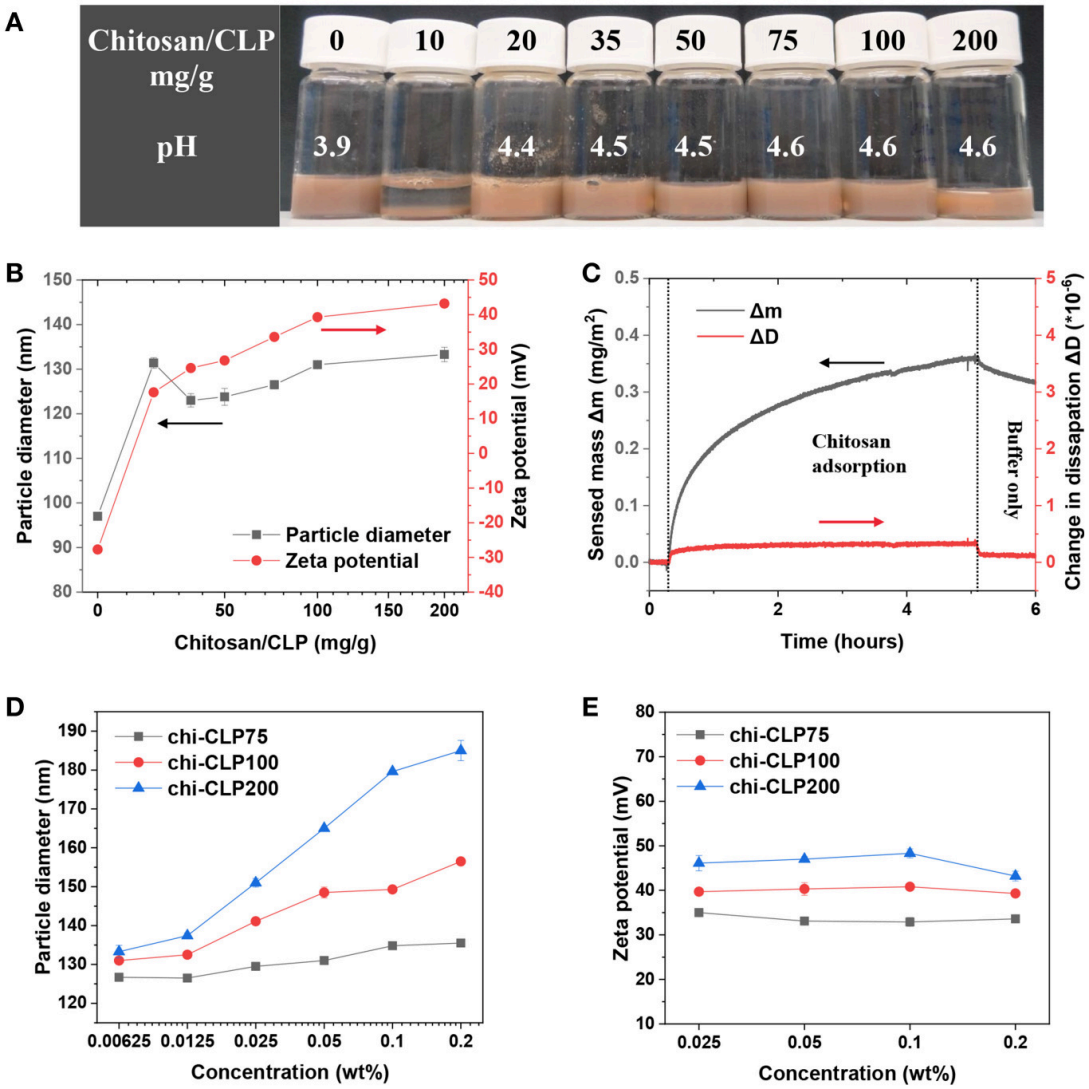
Characterization of coating of CLPs with chitosan. **(A)** Photograph of the chi-CLP dispersions (0.2 wt%, with chitosan-to-CLP mass ratio varying from 0 to 200 mg/g) after storage for 2 h. **(B)** Particle diameter and zeta potential of chi-CLPs plotted against the mass ratio of chitosan to CLP. **(C)** QCM-D analysis of adsorbed mass of chitosan on lignin surface. **(D)** Particle diameter of chi-CLP75, chi-CLP100, and chi-CLP200 plotted against the chi-CLP concentration, chi-CLP dispersions were diluted with pH 4.5 aqueous acetic acid. **(E)** Zeta potential of chi-CLP75, chi-CLP100, and chi-CLP200 plotted against chi-CLP concentration, chi-CLP dispersions were diluted with pH 4.5 aqueous acetic acid. The error bars in **(B,D,E)** denote the standard deviations of three replicates. Note: chi-CLP10 was not measurable by DLS due to strong aggregation and sedimentation.

The CLPs purified by dialysis (0.2 wt%) were used for assessing the effect of mass ratio of chitosan to CLP on the particle properties. It was determined by DLS that pure CLPs had the average particle diameter of 97 nm (PDI 0.18) and zeta potential of −27 mV (at native pH 3.9). When 10 mg/g chitosan to CLP was added into the CLP dispersion, the dispersion showed strong aggregation and sedimentation, indicating neutralization of CLPs by chitosan (Figure 2A). When 20 mg/g chitosan to CLP was added, the zeta potential of CLP was reversed from negative (−27 mV) to positive (17.6 mV), showing overcompensation of the surface charge by chitosan. The overcompensation can be explained as follows. Firstly, chitosan could easily adsorb onto the surfaces of CLPs, which was mainly driven by the large entropy gain of the released counterions from both CLPs and chitosan (Kronberg et al., 2014). Secondly, once adsorbed, columbic attraction, hydrogen bonding and van der Waals forces were the attraction forces between chitosan and CLP. The adsorption also caused conformational entropy loss of chitosan, yet this was much smaller relative to the entropy gain of the released counterions. On the basis of adsorption, the overcompensation could happen as the absolute charge density of chitosan (apparent zeta potential +54.4 mV) (Figure S2) was higher than that of CLPs (−27 mV). However, at the mass ratio 20 mg/g chitosan to CLP, the amount of chitosan was not sufficiently high to cover all the surfaces of CLPs, which was indicated by the broad particle diameter distribution and not high enough positive surface charge of chi-CLP20 (Figure S3).

At the mass ratio of 35 mg/g chitosan to CLP, the chi-CLP35 exhibited a higher surface charge density (24.6 mV) than that of chi-CLP20 (17.6 mV), and the resulting particle diameter distribution of chi-CLP35 (PDI 0.25) was also much narrower than that of chi-CLP20 (PDI 0.43) (Figure 2B and Figure S3). Those values indicated that, at 35 mg/g, the CLPs were efficiently covered by chitosan, and thus the chi-CLP dispersion became stable. Higher mass ratios of chitosan to CLP resulted in similar particle diameter distributions (Figure S3) compared to that obtained at 35 mg/g, but a slight increase in the particle diameter as well as zeta potential was observed (Figure 2B). Such observation resulted from the presence of excess chitosan in the aqueous phase. With excess chitosan relative to the CLP surfaces, large chitosan molecules were more prone to adsorb than the smaller ones due to lower solubility/stability of large chitosan molecules in solution. This phenomenon has been described in detail elsewhere (Fu and Santore, 1998; Terada et al., 2004; Kronberg et al., 2014). The adsorption of chitosan to lignin surface was also studied by using QCM-D. It was shown in Figure 2C that, the adsorption of chitosan on lignin film was rather slow, probably due to the low negative charge of lignin. Comparing to CLPs that formed via self-assembly, lignin film that was prepared by spin-coating probably had a lower density of charged groups oriented toward the aqueous phase. In addition, the low value of sensed mass Δ*m* and negligible change in dissipation factor ΔD detected by QCM-D indicated that the adsorbed chitosan layer on lignin surface was very thin (Rodahl and Kasemo, 1996; Naderi and Claesson, 2006).

The excess of chitosan in the aqueous phase was indicated by DLS measurement. As shown in Figure 2D, the increase of chitosan/CLP mass ratio from 75 to 200 mg/g resulted in a stronger dependency of the particle diameter as the function of chi-CLP concentration. The particle diameter of chi-CLP75 increased slightly with increased concentration, yet it increased significantly more in the case of chi-CLP200. Such phenomena can be explained by the increased viscosity of the aqueous media in the presence of excess chitosan. The more excess chitosan in the aqueous phase, the larger the overestimation of particle diameter determined by DLS. The swelling/deswelling of chi-CLPs could be neglected in this case, as the dispersions were diluted with pH 4.5 acetic acid and thus the pH and ionic strength remained constant throughout the experiments. As another evidence, the zeta potential remained essentially unchanged as a function of chi-CLP concentration (Figure 2E). The unchanged zeta potential also indicated that no detectable desorption of chitosan occurred upon dilution.

### Effect of Chitosan to CLP Mass Ratio on Emulsion Formation

**Figure 3:**
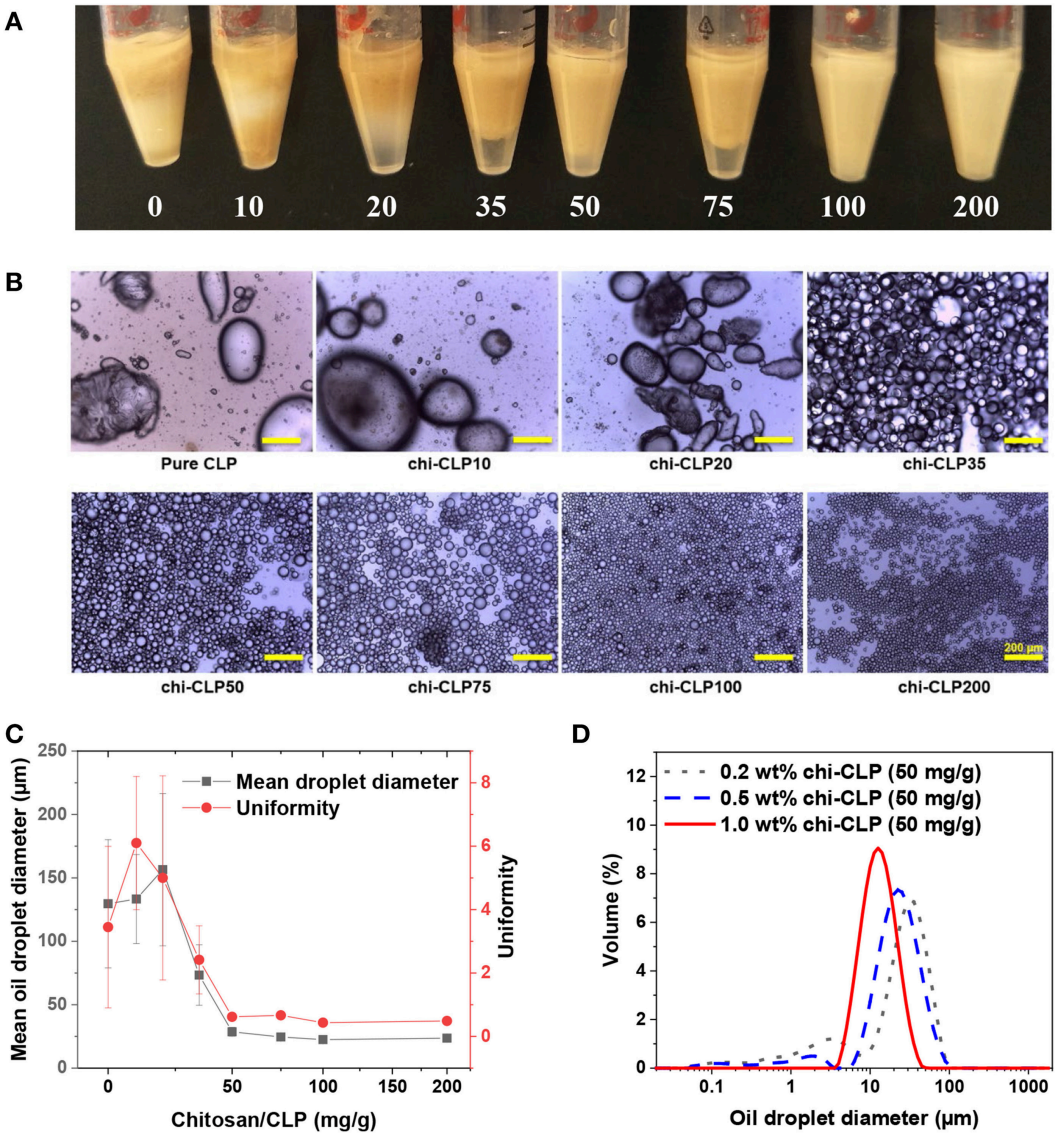
Effect of chitosan to CLP mass ratio and chi-CLP concentration on the properties of olive oil-in-water (volume ratio 1:1) Pickering emulsions (formed by ultrasonication). **(A)** Photograph of the Pickering emulsions stabilized by 0.2 wt% chi-CLPs (0 to 200 mg/g). **(B)** Optical microscopic images of the Pickering emulsions stabilized by 0.2 wt% chi-CLPs (0 to 200 mg/g) (Scale bars: 200 μm). **(C)** Mean oil droplet diameter (d_43_) and uniformity (according to Equation 1) plotted against the mass ratio of chitosan to CLP from 0 to 200 mg/g (0.2 wt%). The error bars denote standard deviations of six replicates. **(D)** Diameter distribution of the oil droplets stabilized by 0.2, 0.5, and 1 wt% chi-CLPs at a fixed mass ratio of 50 mg/g chitosan to CLP. Note: photograph, optical microscopic images and diameter measurements were done 24 h after emulsion formation.

Olive oil-based Pickering emulsions were formed using 0.2 wt% chi-CLP dispersions, in which the mass ratio of chitosan to CLP varied from 0 to 200 mg/g. The volume ratio of olive oil to chi-CLP dispersion was fixed at 1:1. The emulsions were formed by ultrasonication and hand-shaking. It was found that, the mass ratio of chitosan to CLP had a strong effect on the properties of the emulsions that formed by ultrasonication. At low mass ratio of chitosan to CLP (≤ 20 mg/g), the formed emulsions were poor that showed broad biphasic diameter distributions of the oil droplets (Figure 3B and Figure S4). However, with a higher mass ratio of chitosan to CLP at 35 mg/g, a strong increase of the fraction of small oil droplets with diameter between ca. 10 to 100 μm and reduction in droplets between 100 to 1,000 μm occurred (Figure S4). Effectively, optical microscopic images showed that oil droplets from around 10 to 100 μm dominated the diameter distribution (Figure 3B). This transition happened in accordance with the partial to efficient coverage of CLPs by chitosan from 20 to 35 mg/g mass ratios. We can therefore conclude that a sufficient coverage of CLPs by chitosan is essential for stabilization of olive oil in water. This finding correlates well with the limited emulsification capacity of regular anionic CLPs (Sipponen et al., 2017) and good emulsification capacity of cationic chitosan for triglycerides (Schulz et al., 1998; Del Blanco et al., 1999; Rodriǵuez et al., 2002). When the mass ratio of chitosan to CLP was increased to 50 mg/g, the distribution of the droplets became nearly monophasic, the droplets mainly distributed between 10 to 100 μm and the “macro” droplets from 100 to 1,000 μm vanished (Figure S4A). Such transition can be explained from two aspects. On the one hand, the cationic charge density along with the hydrophilicity (Zhang S. et al., 2015) of the chi-CLPs increased with higher chitosan-to-CLP coating ratio, which enhanced the attraction of chi-CLP to olive oil that contained 2 wt% of negatively charged oleic acids. On the other hand, the excess chitosan in the aqueous phase was likely involved in forming the emulsion with chi-CLP and thus reduced the coalescence of the oil droplets. The involvement of chitosan in the emulsion formation was also indicated by the fact that the average diameter of oil droplets decreased slightly further with more excess chitosan from chi-CLP50 to chi-CLP200 (Figures 3B,C). The mean droplet diameter nearly plateaued at ~25 μm, which is considerably smaller size than reported for triglyceride-in-water Pickering emulsions stabilized with chitosan particles alone or as mixtures with STP (Mwangi et al., 2016a; Shah et al., 2016).

Comparison of the amounts of chi-CLPs required to obtain stable emulsions to those in the literature reveals that the emulsifier-to-oil ratios obtained in the present work are significantly lower. The stable emulsions in Figure 3A contained chi-CLPs at the coating ratio of 100 mg/g and at 0.2 wt% concentration that equals 0.2 wt% emulsifier relative to olive oil. Previously, 0.5 wt% of cationic lignin-coated CLPs were used in incomplete stabilization of olive oil/water emulsion (Sipponen et al., 2017), while the values for aggregated chitosan particles range from 0.3 wt% (oleic acid-in-water) (Asfour et al., 2017) to 1.2 wt% (medium chain triglycerides-in-water) (Mwangi et al., 2016b). We expect that the lower ratio of chi-CLPs that suffices for emulsion stabilization in the present work stems from the large surface area and efficient assembly of the colloidal particles at the oil/water interface.

Interestingly, when comparing the emulsions that formed by hand-shaking. Even chi-CLP10 succeeded in forming the emulsion with olive oil, while pure CLP failed to do so. Additionally, optical microscopic images showed that all the emulsions showed broad droplet diameter distributions ranging from a few to hundreds micrometers regardless of chitosan to CLP mass ratio (Figure S5). This is reasonable, because the energy input generated by manual shaking is much lower and inhomogeneous compared to that of ultrasonication. As a consequence, hand-shaking could not produce small and uniform emulsion droplets even with the presence of excess chitosan.

### Effect of chi-CLP Concentration on Emulsion Formation

**Figure 4:**
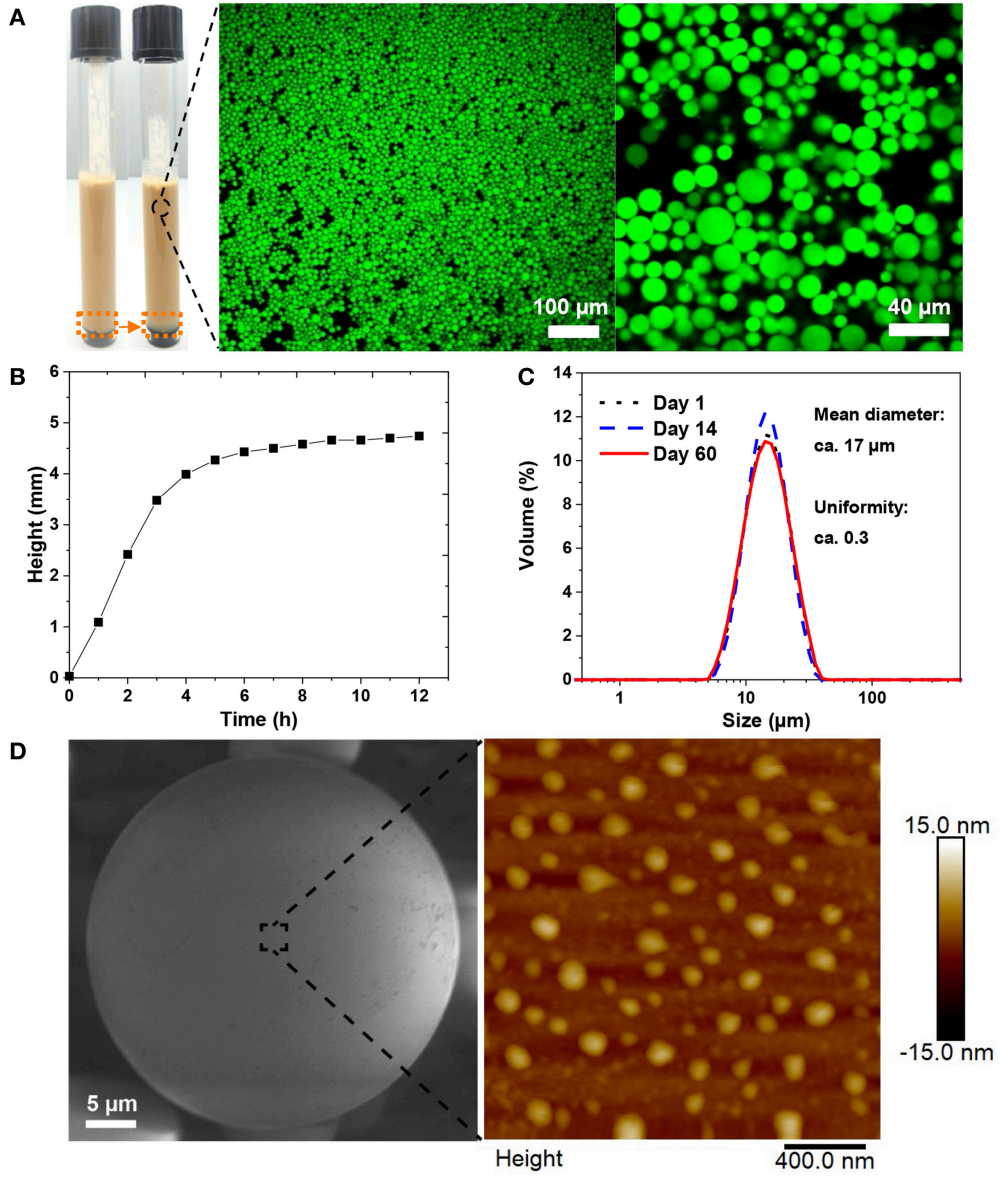
Stability and surface morphology of the emulsion stabilized by 1 wt% chi-CLP (50 mg/g). **(A)** Photograph of the emulsion (before and after creaming) and the corresponding confocal microscopic images of the emulsion (oil was stained by Nile red). **(B)** Creaming behavior of the emulsion, measured with Turbiscan (height was chosen at the backscattered intensity 0.5 (Figure S9) and rescaled by starting from 0, corresponding to the height change marked by orange dash boxes in **A**). **(C)** Diameter distributions of the oil droplets on day 1, day 14, and day 60 from emulsion formation. **(D)** Surface morphology of a single oil droplet covered by STP cross-linked chi-CLPs, imaged with E-SEM (left, oil was solidified at −20°C) and AFM (right, measured at ambient conditions).

Aforementioned results showed that the minimum coating ratio at which the emulsions became nearly monophasic was 50 mg/g chitosan to CLP. Therefore, this mass ratio was fixed for studying the effect of chi-CLP concentration on the emulsion properties. Three different concentrations of chi-CLP dispersions (0.2, 0.5, and 1 wt% chi-CLP) were used to form emulsions with olive oil (volume ratio 1:1) by ultrasonication. As anticipated, higher concentration of chi-CLP dispersion resulted in emulsions with smaller mean droplet diameter and better uniformity (Figure 3D). This phenomenon can be understood from the perspective of diffusive and adsorptive kinetics of the chi-CLPs. At higher concentration of the chi-CLP dispersion, a shorter diffusion time of the particles to the oil/water interfaces and a faster adsorption occurs due to shorter diffusion distances. As a consequence, the oil droplets were more efficiently and rapidly covered by the chi-CLPs during the transient mixture of oil and water caused by ultrasonication, which therefore resulted in less coalescence of the oil droplets during formation. Finally, an emulsion with smaller and more uniform droplets was obtained. At the highest concentration (1 wt%) of chi-CLP dispersion, the formed emulsion showed the smallest mean droplet diameter of ca. Seventeen micrometer with the most uniform diameter distribution (Figures 3D, 4A). Such uniformity value of ca. 0.3 outperforms the uniformity of 0.5 reported for palm oil/water Pickering emulsion stabilized by aggregated chitosan particles (Mwangi et al., 2016a). As another comparison, chitosan molecule alone could not result in such uniform droplets with olive oil, even at the concentration of 1 wt% (Figure S6). Furthermore, long term observation (over 6 months) found that the emulsions stabilized by chitosan molecules were much less stable than those stabilized by chi-CLPs (Figure S7). Additionally, calculated from the data in Figure 4A, the emulsion stability index (volume ratio of the emulsion layer to the total volume) was nearly one, which is obviously much higher than that obtained with cationic colloidal lignin particles (Sipponen et al., 2017).

### Stability and Surface Morphology of the Emulsion

The emulsion stabilized by 1 wt% chi-CLP (50 mg/g) was selected for further stability test. In general, the emulsion exhibited a strong stability against coalescence, where the droplet diameter distribution of the emulsion did not change over 2 months (Figure 4C). Such strong stability resulted from the high energy requirement for the desorption of chi-CLPs from oil/water interface and the positive charge of the chi-CLPs that providing electrostatic stabilization (Binks and Clint, 2002; Rayner et al., 2014). In addition, the relative high viscosity (Figure 4B) also contributed to the high stability of the emulsion. As a consequence, the emulsion could be utilized for drug storage purposes, since many drugs are degradable when exposed to oxygen and UV light, which can be inhibited by the antioxidative, and UV shielding properties of the lignin nanoparticles (Farooq et al., 2019).

The surface morphology of the capsule was rather smooth when observed under environmental SEM (Figure 4D, left). A closer observation by AFM showed that the particles were partly embedded in the oil droplets (Figure 4D, right). SEM also showed that a longer exposure of the frozen droplet to the electron beam could expose the embedded chi-CLPs from the surface of the oil droplet (Figure S8). Such observations indicated that the chi-CLPs had a strong affinity to the oil.

### Ionic Cross-Linking of the Emulsion Capsules for Enhanced Mechanical Performance

**Figure 5:**
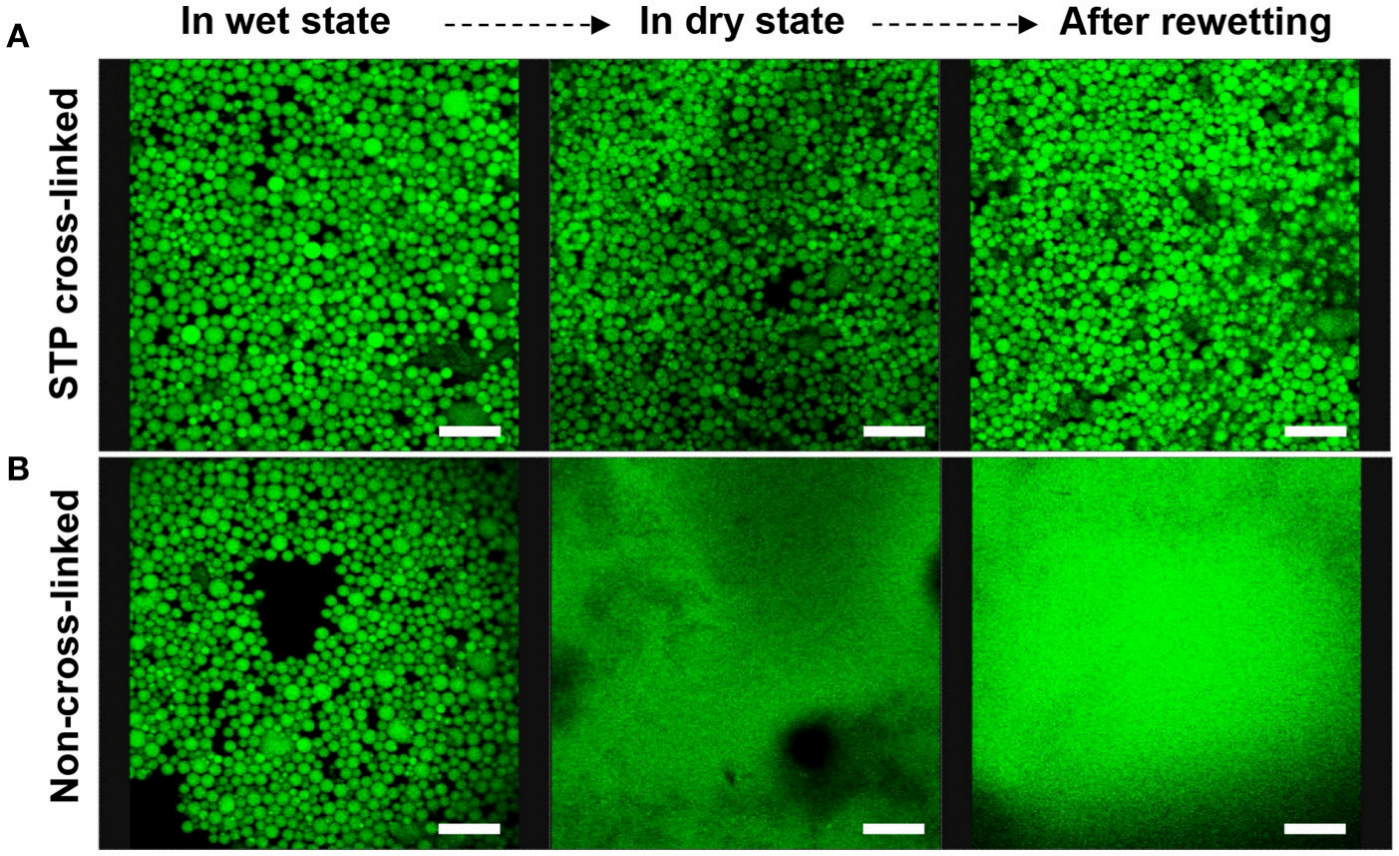
Confocal microscopic images of sodium triphosphate (STP) cross-linked and non-cross-linked olive oil-in-water (volume ratio 1:1) Pickering emulsions stabilized by 1 wt% chi-CLP (50 mg/g) (Scale bars: 80 μm). **(A)** STP cross-linked emulsion in wet state (oil droplets in water), in dry state (water evaporated at room temperature), and after rewetting with deionized water. **(B)** Non-cross-linked emulsion in wet state, dry state and after rewetting as the reference.

The emulsion stabilized by 1 wt% chi-CLP (50 mg/g) was selected for the ionic cross-linking study. Instead of covalent cross-linking of chi-CLPs, ionic cross-linking is reversible, and can be done easily at ambient conditions. In this work, sodium triphosphate (STP) was used because it has low toxicity (Human and Environmental Risk Assessment, CAS: 7758-29-4)^1^ and has been used successfully in cross-linking chitosan (Mwangi et al., 2016a; Shah et al., 2016; Larbi-Bouamrane et al., 2017). The ionic cross-linking of chi-CLP by STP followed similar mechanism as the CLP coating by chitosan. Briefly, the replacement of the monovalent counterions of chitosan by trivalent triphosphate resulted in a large entropy gain. After cross-linking of the emulsion capsules, the mechanical stability of the oil droplets increased significantly. The oil could still be retained in the chi-CLP capsules even after drying and rewetting, indicating that the corona of the capsules was very strong (Figure 5A). In comparison, the non-cross-linked emulsion capsules easily broke down after drying and released the oil (Figure 5B). Similar observation was confirmed by optical microscopy, which showed that the cross-linked emulsion capsules retained their round shape after drying, yet the non-cross-linked ones were broken up to irregular shapes (Figure S10). With the exception of organic/inorganic capsules prepared by emulsion polymerization (Zhang et al., 2010; Wang et al., 2012), essentially all of the prior works have visualized shell rupture of Pickering capsules upon drying (van Rijn et al., 2011; Sipponen et al., 2017). In addition to their enhanced stability, the rewettable emulsion capsules prepared in the current work enable changing the aqueous phase or forced diffusion of oleophilic substances into the capsules by water evaporation. Besides, compared to the covalent epoxy cross-linking of lignin capsules demonstrated by Tortora et al. (2014), our approach is reversible. The ionic cross-linkers are released into aqueous phase upon dilution by water due to the deprotonation of the protonated primary amine groups (pK_a_ 6.5), which is beneficial for dissembling of the capsules that serve as drug carriers.

### Ciprofloxacin Release From the Emulsion Capsules at Various PH Values

**Figure 6:**
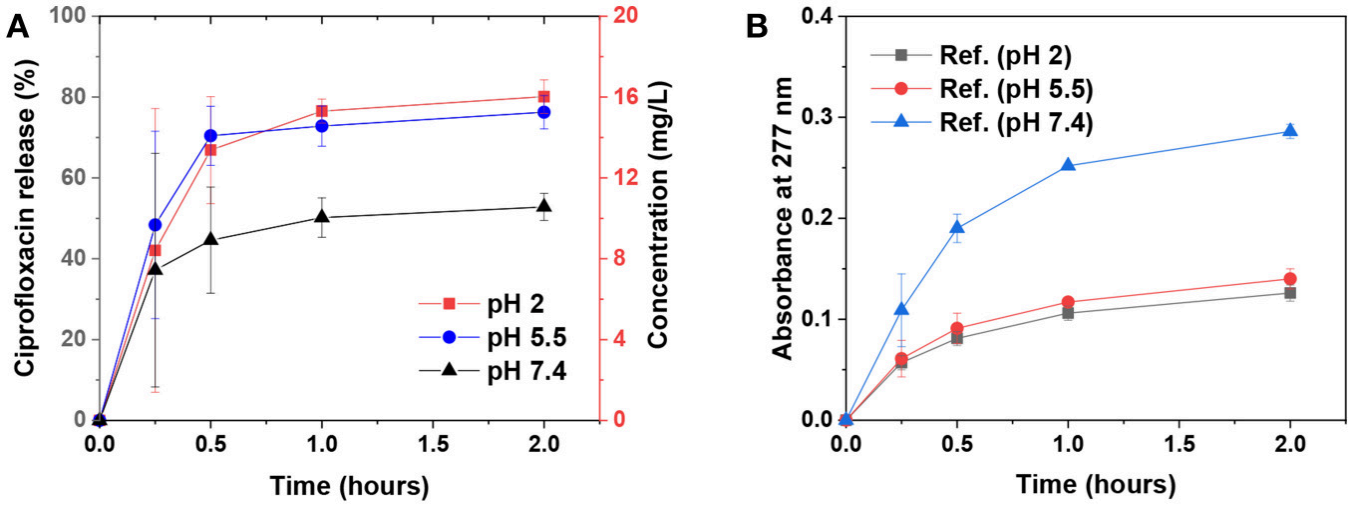
Ciprofloxacin release from the emulsion capsules [stabilized by 1 wt% chi-CLP (50 mg/g)] to the buffer solutions at various pH values. **(A)** Ciprofloxacin release kinetics at pH 2, 5.5, and 7.4 at 37°C, mean values ± standard deviations of four replicate samples are shown. **(B)** Absorbance of the reference samples (without ciprofloxacin) at 277 nm measured under same conditions as the ciprofloxacin-loaded samples, mean values ± absolute deviations of two replicates are shown.

The biocompatibility of various types of lignins is currently under intense investigation. Recent studies have indicated that kraft lignin and alkali lignin are not antiproliferative and instead mostly biocompatible (Tortora et al., 2014; Dai et al., 2017; Figueiredo et al., 2017). We selected ciprofloxacin as a model drug for the release demonstration by the emulsion capsule which was stabilized by 1 wt% chi-CLP (50 mg/g). Ciprofloxacin is an antibiotic drug (Zeiler and Grohe, 1986) that has potential synergic effect with chi-CLPs that likely possess antimicrobial activities (Croisier and Jérôme, 2013; Beisl et al., 2017). The drug release experiment was conducted under simulated physiological conditions, i.e., at pH 2, 5.5, and 7.4 at 37°C. As shown in Figure 6A, the release rate of ciprofloxacin from the capsule to the buffer was rather fast regardless of the pH. The leveling-off concentration was lower at pH 7.4 compared to that of pH 2 and 5.5, mainly because lower aqueous solubility of the drug at higher pH (Maurer et al., 1998). The fast release was related to the small diameter of the droplets, which were well-dispersed in the buffer at the initial stage. Additionally, the chi-CLP coating of the oil droplets allowed rapid diffusion and leakage of the drug from inter-particle pores. Apart from that, the reference samples (without ciprofloxacin) showed that the emulsion capsules were much more stable at acid pH compared to pH 7.4, where the absorbance at 277 nm indicated partial dissolution of CLPs (Figure 6B). From this perspective, the microcapsules can be beneficial for an intestinal drug delivery, since the dissolution of CLPs at higher pH can result in a larger porosity of the capsules and thus faster drug release.

## Conclusion

In this study, the important parameters for the preparation of colloidally stable chitosan-coated CLPs and their application for emulsion stabilization were studied. We found that the thin film coating of CLPs by chitosan significantly improved their emulsification capacity for olive oil. In addition, the uniformity of the emulsion droplets could be further improved at a higher concentration of chitosan-coated CLP (chi-CLP) dispersion. The obtained Pickering emulsions exhibited strong stability against coalescence, and the droplet diameter distribution remained almost unchanged for over 2 months. The positively charged chi-CLP layer locating at the oil/water interface could be electrostatically cross-linked by sodium triphosphate, which resulted in enhanced mechanical stability of the emulsion capsules. Such microcapsule with the shell that mainly comprised of CLPs are beneficial for instance for drug storage purposes, since CLPs possess antioxidative and UV-shielding properties. On the other hand, the cationic net charge of the microcapsules due to chitosan is also interesting with respect to bioadhesion and drug delivery into cells. Ongoing work in our laboratory is focused on closer investigation of these interactions of colloidal lignin particles with living cells.

## Author Contributions

TZ designed and carried out the experiments under the guidance of MS. The results were analyzed by TZ with input from MS and MÖ. The manuscript was drafted by TZ with contributions from all of the authors.

### Conflict of Interest Statement

The authors declare that the research was conducted in the absence of any commercial or financial relationships that could be construed as a potential conflict of interest. The handling editor declared a shared affiliation, though no other collaboration, with TZ, MS, MÖ at time of review.

## Footnotes

^1^*Human and Environmental Risk Assessment, CAS: 7758-29-4*. Available online at: https://www.heraproject.com/files/13-f-04-%20hera%20stpp%20full%20web%20wd.pdf (accessed December 21, 2018).
