# Supplementary material for: Natural Shape-Retaining Microcapsules With Shells Made of Chitosan-Coated Colloidal Lignin Particles
Source: Front Chem. 2019 May 22;7:370. doi: 10.3389/fchem.2019.00370 (PMC6540915; doi:10.3389/fchem.2019.00370)
Supplement: Supplementary file 1 [file Table_1.pdf]

Supplementary information

# Natural shape-retaining microcapsules with shells made of chitosan-coated colloidal lignin particles

Tao Zou[1], Mika H. Sipponen[1] and Monika Österberg[1]*

[1] Department of Bioproducts and Biosystems, School of Chemical Engineering, Aalto University, Espoo, Finland. * Correspondence: monika.osterberg@aalto.fi

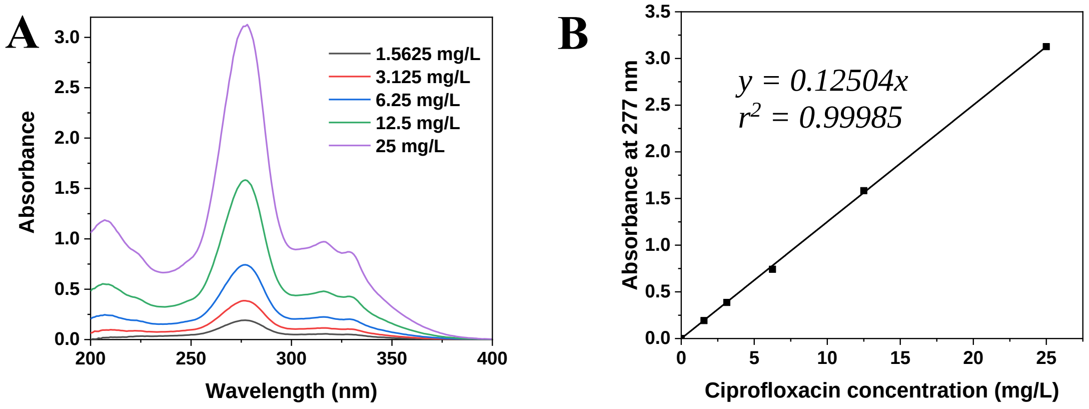


**Figure S1 | (A)** UV-vis spectra of ciprofloxacin in dilute acetic acid solution at various concentrations with the maximum UV absorbance at 277 nm. (**B**) UV absorbance of ciprofloxacin at 277 nm as a function of ciprofloxacin concentration.

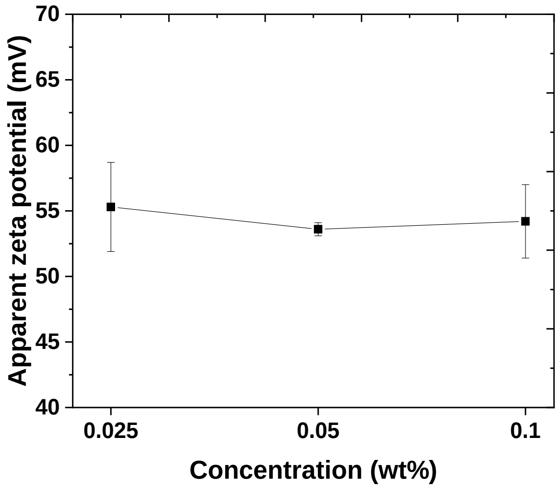


**Figure S2 | Apparent zeta potential of chitosan plotted against the concentration.** 1 wt-% chitosan was firstly dissolved in 0.1 M acetic acid and then diluted with pH 4.5 aqueous acetic acid.

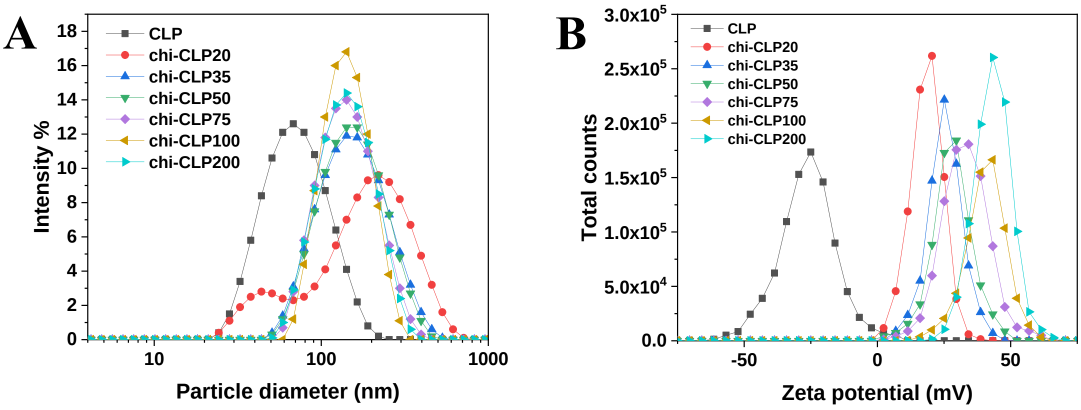


**Figure S3 | (A)** Intensity-based particle diameter distributions of the chi-CLPs (0.2 wt-%) with the mass ratio of chitosan to CLP varying from 0 to 200 mg/g. **(B)** Number-based zeta potential distributions of the chi-CLPs (0.2 wt-%) with the mass ratio of chitosan to CLP varying from 0 to 200 mg/g.

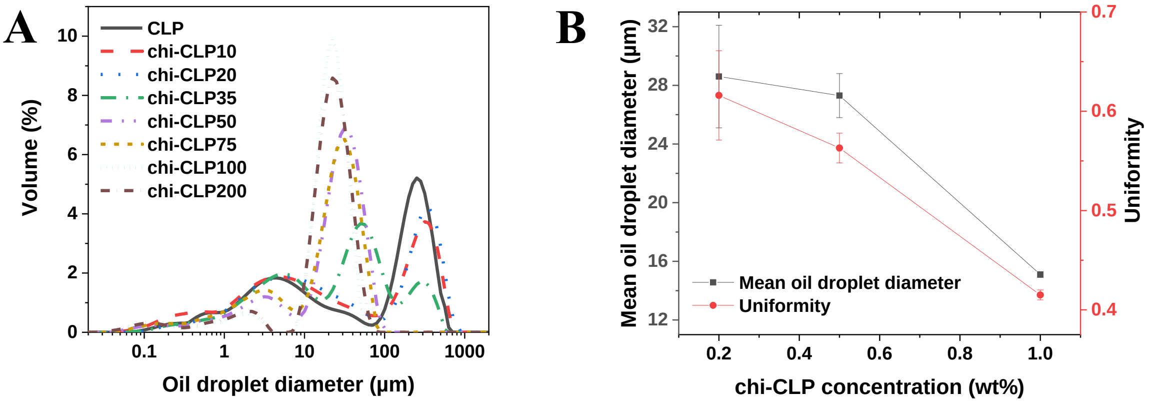


**Figure S4 | (A)** Diameter distributions of the oil droplets stabilized by chi-CLPs (0.2 wt-%) with the mass ratio of chitosan to CLP varying from 0 to 200 mg/g. **(B)** Mean oil droplet diameter ($d_{43}$) and uniformity (according to Eq. 1) of the emulsion plotted against the concentration of chi-CLP (50 mg/g).

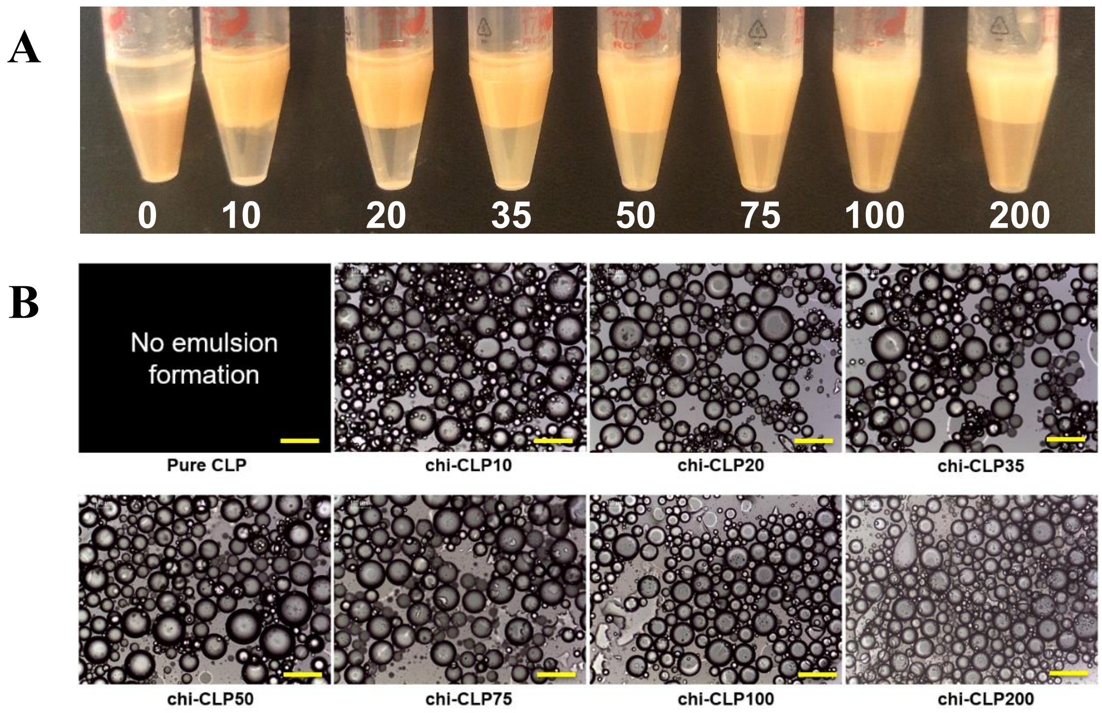


**Figure S5 | Olive oil-in-water (volume ratio 1:1) Pickering emulsions formed by vigorous hand-shaking for 60s. (A)** Photograph of the Pickering emulsions stabilized by chi-CLPs (0.2 wt-%) with the mass ratio of chitosan to CLP varying from 0 to 200 mg/g. **(B)** Optical microscopic images of the Pickering emulsions stabilized by chi-CLPs (0.2 wt-%) with the mass ratio of chitosan to CLP varying from 0 to 200 mg/g (Scale bars: 200 µm).

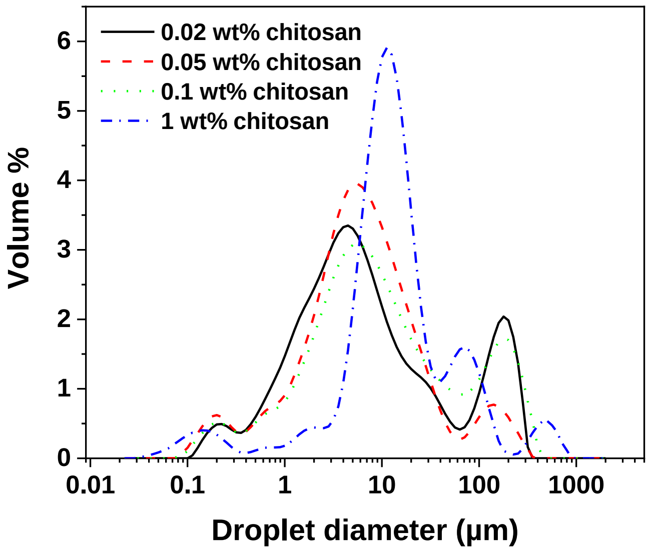


**Figure S6 | Olive oil-in-water emulsions stabilized by chitosan molecules at the concentration varying from 0.02 to 1 wt-%.** Emulsions were formed by ultrasonication and the droplet diameter were measured 3 days after emulsion formation.

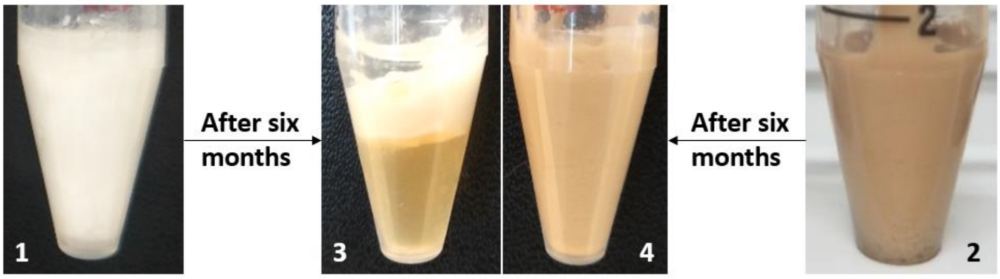


**Figure S7 | Comparison of the stability of the olive oil-in-water (volume ratio 1 : 1) emulsions:** 1 wt-% chitosan-stabilized emulsion on day 0 (1) and after six months (3); 1 wt-% chi-CLP (50 mg/g) stabilized emulsion on day 0 (2) and after six months (4).

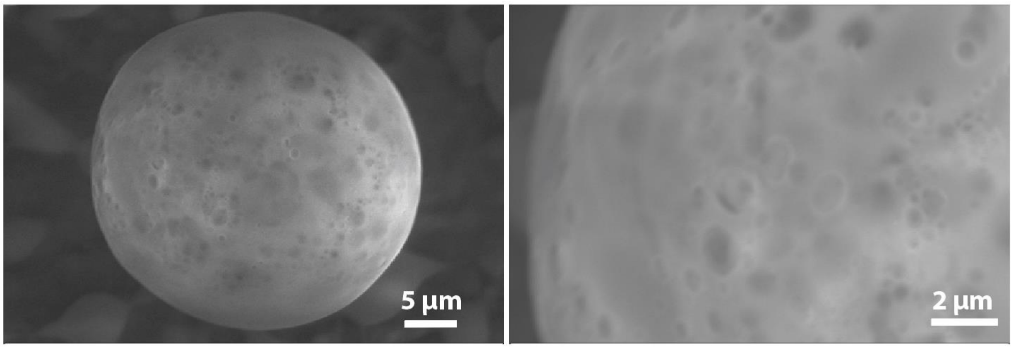


**Figure S8 | Surface morphology of the oil droplet covered by STP cross-linked chi-CLPs (50 mg/g), imaged with E-SEM (oil was solidified at -20 °C).** Images were captured after a prolonged exposure of the Pickering emulsion droplet to the electron beam.

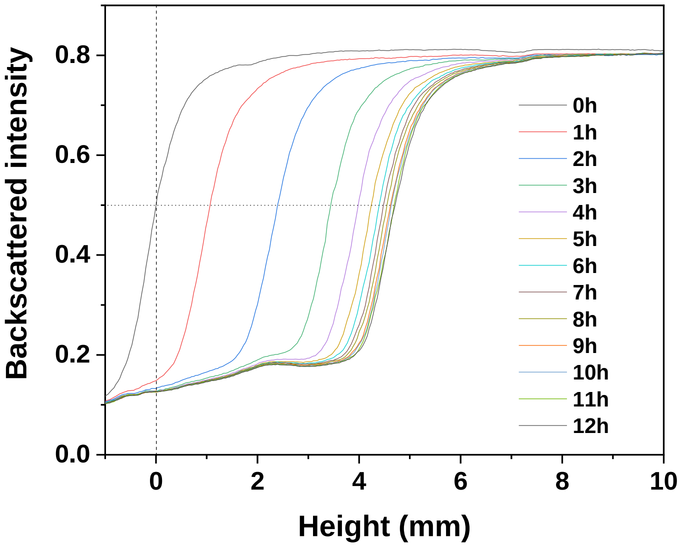


**Figure S9 | Creaming behavior of the Pickering emulsion stabilized by 1 wt-% chi-CLP (50 mg/g), measured with Turbiscan.** Height was rescaled by starting from 0 at the intensity 0.5 (indicated by the dashed lines).

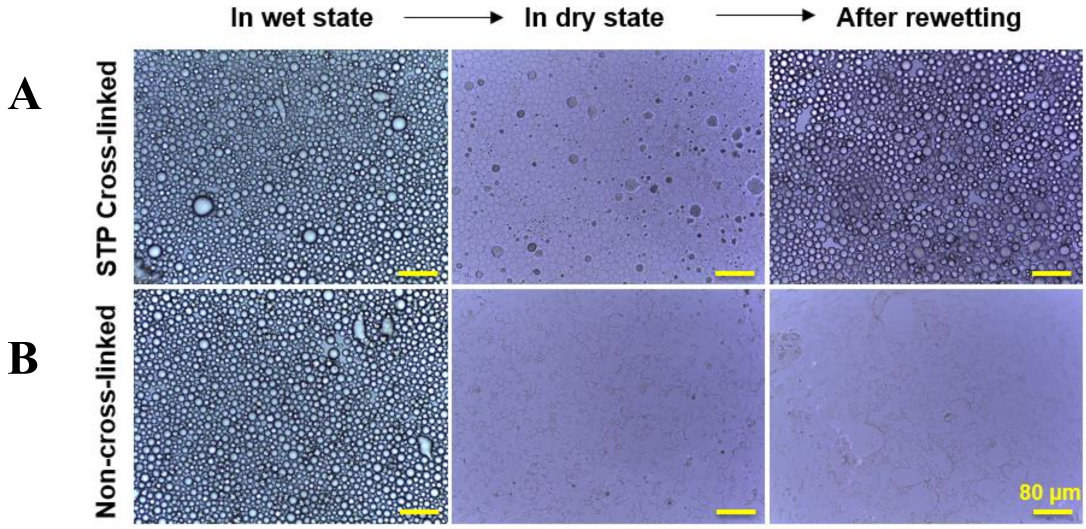


**Figure S10 | Optical microscopic images of sodium triphosphate (STP) cross-linked and non-cross-linked olive oil-in-water (volume ratio 1:1) Pickering emulsions stabilized by 1 wt-% chi-CLP (50 mg/g). (A)** STP cross-linked emulsion in wet state (oil droplets in water), in dry state (water evaporated at room temperature) and after rewetting with deionized water. **(B)** Non-cross-linked emulsion in wet state, dry state and after rewetting as the reference. Scale bars: 80 µm.
